# Landmark tracking in 4D ultrasound using generalized representation learning

**DOI:** 10.1007/s11548-022-02768-z

**Published:** 2022-10-15

**Authors:** Daniel Wulff, Jannis Hagenah, Floris Ernst

**Affiliations:** 1 Institute for Robotics and Cognitive Systems, University of Lübeck, Ratzeburger Allee 160, Lübeck, 23562 Schleswig-Holstein Germany; 2 Department of Engineering Science, Institute of Biomedical Engineering, University of Oxford, ParksRoad, Oxford, OX1 3PJ UK

**Keywords:** Sliced-Wasserstein autoencoder, Greedy search, Radiotherapy, Representation space

## Abstract

**Purpose:**

In this study, we present and validate a novel concept for target tracking in 4D ultrasound. The key idea is to replace image patch similarity metrics by distances in a latent representation. For this, 3D ultrasound patches are mapped into a representation space using sliced-Wasserstein autoencoders.

**Methods:**

A novel target tracking method for 4D ultrasound is presented that performs tracking in a representation space instead of in images space. Sliced-Wasserstein autoencoders are trained in an unsupervised manner which are used to map 3D ultrasound patches into a representation space. The tracking procedure is based on a greedy algorithm approach and measuring distances between representation vectors to relocate the target . The proposed algorithm is validated on an in vivo data set of liver images. Furthermore, three different concepts for training the autoencoder are presented to provide cross-patient generalizability, aiming at minimal training time on data of the individual patient.

**Results:**

Eight annotated 4D ultrasound sequences are used to test the tracking method. Tracking could be performed in all sequences using all autoencoder training approaches. A mean tracking error of 3.23 mm could be achieved using generalized fine-tuned autoencoders. It is shown that using generalized autoencoders and fine-tuning them achieves better tracking results than training subject individual autoencoders.

**Conclusion:**

It could be shown that distances between encoded image patches in a representation space can serve as a meaningful measure of the image patch similarity, even under realistic deformations of the anatomical structure. Based on that, we could validate the proposed tracking algorithm in an in vivo setting. Furthermore, our results indicate that using generalized autoencoders, fine-tuning on only a small number of patches from the individual patient provides promising results.

## Introduction

In recent years, ultrasound imaging has become of high interest for usage in image guided interventions, e.g., in minimally invasive surgery [1] or in radiation therapy [2]. The ability to acquire volumetric images of soft tissue in real-time makes it a promising modality for guidance and tracking. One common problem in precise image guidance is tracking a target structure over time, e.g., an anatomical landmark. Examples range from ultrasound-guided radiotherapy [3] to the autonomous repositioning of the ultrasound probe using a robotic arm [4].

Typically, target tracking is performed in image space by comparing a reference image patch to candidate patches using pixel-based metrics [5]. However, deformations of the anatomical target in subsequent image frames are likely to be non-rigid, and finding the target structure in the case of complex elastic deformations is challenging [6, 7]. These deformations become even more complex to assess in 3D ultrasound . Hence, previously published approaches focussed on tracking in 2D ultrasound image timeseries. Several approaches for performing tracking in 2D ultrasound are proposed in the CLUST challenge [8] but only a few algorithms are proposed for 3D ultrasound. [9] presented a solution using rigid registration schemes on the basis of point sets and [10] proposed the usage of a shape model that is optimized utilizing internal and external displacements. Another approach was presented by Huang et al. [11] where a deep learning approach is used. Three orthogonal 2D slices serve as the input of a convolutional neural network to find the current landmark position. Although this approach is applied to 3D ultrasound, only a 2D convolutional network is used.

**Fig. 1:**
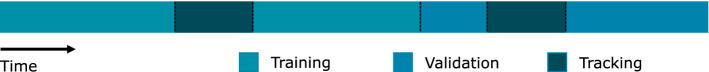
Illustration about how the ultrasound sequences are split into training, validation and tracking sets

All these methods rely on the comparison of candidate patches with a reference patch in image space. In contrast, in Wulff et al. [12], it could be shown that it is possible to learn a deformation-agnostic representation of 3D ultrasound patches utilizing unsupervised representation learning. The identified representation was discriminative regarding different patches but not regarding different elastic deformations of the same patch. This indicates that in this representation, even deformed versions of the target patch should be closer to each other than patches showing other anatomical structures. Therefore, the distance between two patches within this abstract representation might be meaningful measure of the patches similarity, even in the presence of deformations of the target structure.

In this work, we present a novel concept for tracking in 3D ultrasound by assessing the similarity between two image patches in a latent representation derived using unsupervised representation learning. Thus, we formulate a tracking algorithm following this idea and provide a proof of concept on an in vivo data set of liver motion. Furthermore, we present a thorough evaluation of transfer learning concepts to increase the applicability of our method in a clinical scenario.

## Methods

The objective of this study is to investigate whether tracking of deformable objects in time resolved 3D ultrasound can be performed in a representation space. Ultrasound patches are mapped into latent representation vectors, so measuring patch similarities becomes measuring vector distances. A long-term 3D ultrasound data set is used for training and validation of different autoencdoers and for evaluating the tracking performance. It is investigated whether generalized autoencoders are applicable instead of patient individual autoencoders. For the clinical workflow, this would be beneficial and time saving as learning patient individual networks could be replaced by using pretrained generalized networks. However, the optimal representation space dimensionality is unknown yet, so investigating this is part of this study. Ngoc and Hwang [13] proposed to choose the representation space dimensionality on the basis of the task performance. Following this, tracking is performed in different representation space sizes. In this section, the ultrasound data set, the autoencoders and the experiments are introduced.

### Ultrasound data

The ultrasound data used in this study were acquired and published by Ipsen et al. [14]. The data set contains five long-term 3D ultrasound sequences of the liver of five different subjects $S_i$ where $i \in I=\{1,2,3,4,5\}$. In addition to the ultrasound data, landmark annotations are provided. In each data set, two short-term sequences $L_{j, i}$, $j \in \{1,2\}$ with a duration of 30 s each are labeled where one landmark was annotated in every second frame by one expert. These landmark annotations serve as ground truth for the tracking experiments in this study. However, the landmarks in the data set $S_5$ were set quite close to the border of the ultrasound volume. This means that in some parts of the landmark neighborhood no image information is available. Since the proposed tracking method is based on this neighborhood information, the tracking method cannot be applied to this data set. Therefore, the data set $S_5$ is used for training, but the sequences $L_{1\text {-}2, 5}$ are excluded from the tracking experiments. For training and validation, all sequences are split as illustrated in Fig. 1. In the training process, the first two thirds of the sequences are considered (training phase) and for validation the last third is used (validation phase). Note that the labeled sequences $L_{j, i}$ are only used for the tracking experiments and not for training or validation.

**Fig. 2:**
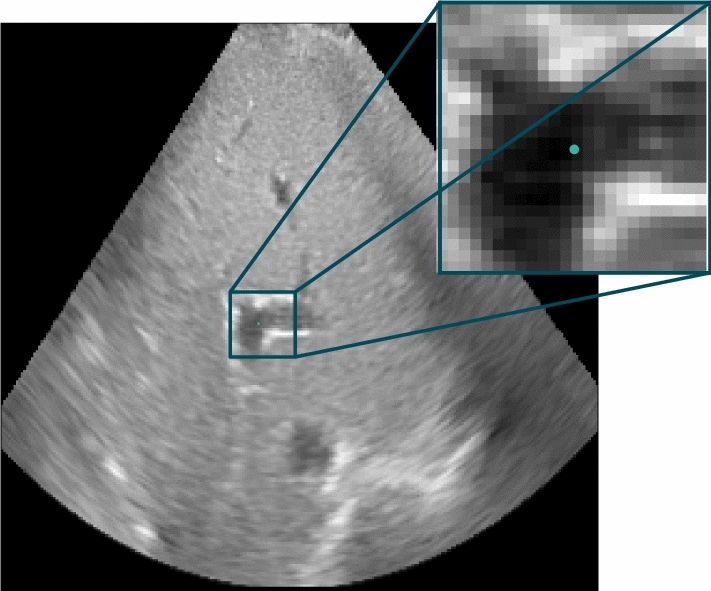
2D slices of an 3D ultrasound volume (left) and a 3D patch (right) selected at the position of an annotated landmark in the data set of subject $S_1$

The autoencoders receive an ultrasound patch in the size $24 \times 24 \times 24$ containing 13,824 voxels as input. In Figure 2, slices of an 3D ultrasound image and an annotated reference patch of $S_1$ are illustrated. For the training process, 50,000 training patches are generated from each training phase $(T_i)$ and for the validation process 5000 patches are similarly generated from each validation phase $(V_i)$. Both the volume in a sequence and the position in the volume where a patch is taken from are selected randomly. Thus, the data sets contain patches with high structure content like vessels as well as patches with less structure such as homogeneous tissue. The data sets that are generated and used in this study are summarized in Table 1.

### Autoencoder architecture

**Table 1:** Ultrasound data sets used for autoencoder training and validation as well as for tracking

Name	Usage	Description
$T_i$	AE training	US patches from training phase
$V_i$	AE validation	US patches form validation phase
$L_{j, i}$	Landmark tracking	US sequences with landmark annotations

**Fig. 3:**
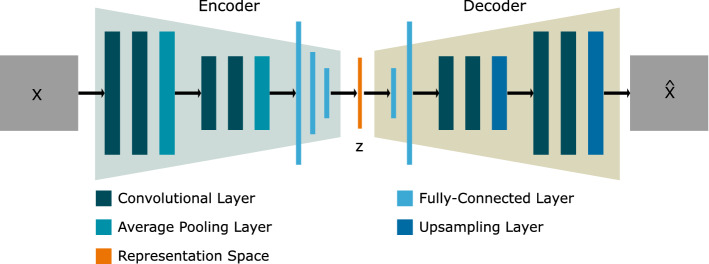
General architecture of the autoencoders. The encoder consists of four convolutional, two average pooling and three fully connected layers, and the decoder consists of four convolutional, two upsampling and two fully connected layers. Several autoencoders with different representation spaces $z \in \mathbb {R}^k$ are used

By using an autoencoder, the tracking process is transferred from the image space into a latent representation space. 3D ultrasound patches are encoded into latent representation vectors using 3D convolutional autoencoders. Thus, the data dimensionality is strongly reduced, while simultaneously the most important information is preserved. The general architecture of the autoencoders is illustrated in Fig. 3. An autoencoder consists of an encoder $q(z\vert X)$ which encodes the ultrasound patch $X \in \mathbb {R}^{24\times 24\times 24}$ into a latent representation vector $z \in \mathbb {R}^{k}$ and a decoder $p\big (\hat{X}\vert z\big )$ that predicts the ultrasound patch reconstruction $\hat{X} \in \mathbb {R}^{24\times 24\times 24}$. The used architectures of the encoder and decoder are determined empirically. The encoder consists of four convolutional layers, two average pooling layers and three fully connected layers. The decoder consists of four convolutional layers, two upsampling layers and two fully connected layers. The autoencoder used in this study is a sliced-Wasserstein autoencoder (SWAE) [15]. During the training process, the SWAE learns to spread all samples into a representation space at a certain distribution. Here, the SWAE forms a hyperbullet-shaped representation space. In contrast to the variational autoencoder (VAE) [16], the SWAE does not use the Kullback–Leibler divergence but the sliced-Wasserstein distance in the loss function. In addition, the mean squared error is used in the loss function to minimize the difference between *X* and ${\hat{X}}$.

1 $$\begin{aligned} \text {Loss}=\alpha \cdot \text {MSE}(X,\hat{X}) + \beta \cdot \text {SW}(p_z, p_\gamma ) \end{aligned}$$

The loss function is given in Eq. (1) where MSE is the mean squared error, SW the sliced-Wasserstein distance [15], and $p_z$ and $p_\gamma$ are the distribution in representation space and the defined distribution, respectively. $\alpha$ and $\beta$ are parameters to weight the loss parts.

### Autoencoder training

Several autoencoders are generated in three different ways. Sets of subject individual autoencoders $(AE_{indiv, i})$, subject generalized autoencoders $(AE_{gen, i}$) as well as generalized fine-tuned autoencoders $(AE_{genFT, i})$ are trained. Each way of training is performed for all subjects $S_i$ and for seven different representation space sizes $k = \{8,16,32,64,128,256,512\}$ each. Reducing the 3D ultrasound patches into the representation space sizes *k* corresponds to a data reduction by $99.94-96.30\%$. Training is performed with the training data sets $T_i$, adam optimizer, a batchsize of 32 and early stopping to avoid overfitting. For this, a validation split of $5\%$ is chosen. The autoencoders $AE_{indiv, i}$ are trained with one data set of $T_i$ each. Autoencoders $AE_{gen, i}$ are trained with four out of five data sets of $T_i$ so that subject generalized autoencoders are generated. In the last step, these autoencoders are fine tuned with 5000 ultrasound patches of the remaining data set of $T_i$. In the fine-tuning process, only the fully connected layers are updated. Table 2 gives an overview about all trained autoencoders.

**Table 2:** Overview of all trained autoencoders

Approach	Autoencoder	Training	Validation	Tracking
		data	data	sequences
Individual	$AE_{indiv, i}$	$T_i$	$V_i$	$L_{j, i}$
Generalized	$AE_{gen, i}$	$T_{h}, h \in I \backslash i$	$V_i$	$L_{j, i}$
Generalized fine-tuned	$AE_{genFT, i}$	$AE_{gen, i}$ + $T_i$ $^{1}$	$V_i$	$L_{j, i}$

$i \in I=\{1,2,3,4\}$ define the subject numbers, and $j \in \{1,2\}$ defines the short-term number

$^{1}$Fine-tuning is performed only with 5000 patches selected randomly from the data set

To evaluate the training success, the reconstruction accuracy is measured using the validation data sets $V_i$. For this, the normalized cross correlation (NCC) given in Eq. (2) is used as metric where *a*, *b* are 3D ultrasound patches and *x*, *y*, *z* are the axes of the 3D ultrasound image space:

2 $$\begin{aligned} \text {NCC}(a,b) = \dfrac{\sum _{x,y,z}{(a(x,y,z)-\bar{a})(b(x,y,z)-\bar{b})} }{\sqrt{\sum _{x,y,z}{(a(x,y,z)-\bar{a})^2} \sum _{x,y,z}{(b(x,y,z)-\bar{b})^2}}} \end{aligned}$$

### Landmark tracking

Using representation learning, the tracking process is transferred from image space into representation space. However, an algorithm to recognize the target representation within a new ultrasound volume is still necessary. A naive approach is using template matching where the target representation is compared to all representation candidates within a region-of-interest [5]. Since this approach is time consuming, especially in 3D data, another approach is used. Due to the slow and continuous motion pattern of the liver tissue, the next target position should be nearby the previous position. Thus, the next position is located using an iterative greedy search starting at the previous position. The algorithm is presented in Algorithm 1. All patches in the three-dimensional 12-neighborhood of the current position are encoded, and the representations are compared to the target representation. If there is a representation in the local neighborhood that is closer to the target representation than the current one, the position is updated. This procedure is repeated until the local optimum is obtained. The distance between two representations is measured using the L2-norm, so the greedy search is looking for the local minimum. 
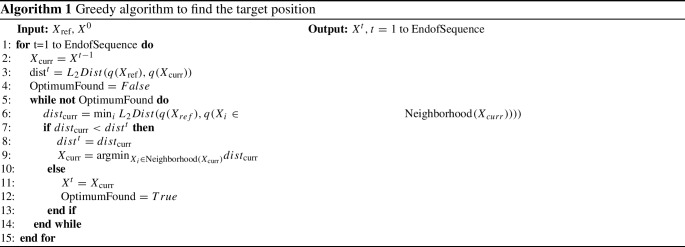
 Liver motion is mainly caused by the breathing cycle and has translation, rotation and deformation parts [6]. Deformation turns tracking into a challenging task due to changes of the target shape and appearance over time. This leads to the fact that the target shape in an inhale phase can highly differ from the target shape in an exhale phase. Thus, four target patches are defined for tracking in this study. The target patches are chosen manually in four different breathing cycle phases. The first and second patch are taken after an inhale and exhale phase, respectively, and the third and fourth patch are taken during an inhale and exhale phase, respectively. Target patches are taken from the other short-term sequence of the same subject. This means, the target patches for tracking in $L_{1, i}$ are taken from $L_{2, i}$ and vice versa. During the tracking procedure (Algorithm 1), the distance between the neighborhood candidates and all target references is measured in the representation space. The neighborhood candidate with the smallest distance to any of the four target references is selected.

All autoencoders are used to perform landmark tracking in the sequences $L_{j, i}$ as illustrated in Table 2. Note that each autoencoder is generated at seven different representation space sizes. The tracking accuracy is evaluated using the ground truth landmarks that are provided with the ultrasound data. In frames where a landmark exists, the L2-norm between the tracking position and the ground truth landmark is measured.

## Results

**Fig. 4:**
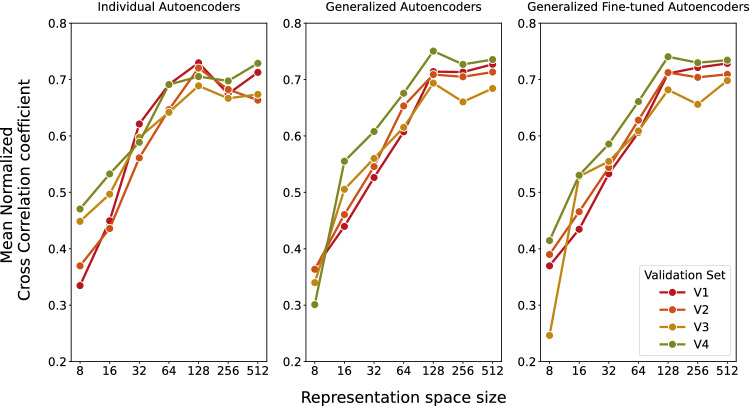
Reconstruction accuracy of the autoencoders $AE_{indiv, i}$ (left), $AE_{gen, i}$ (middle) and $AE_{genFT, i}$ (right) with different representation space sizes

To evaluate the reconstruction accuracy, the autoencoders are tested using the validation data sets $V_i$. The validation data is encoded and decoded, and the reconstructed data is compared to the input data using NCC. The mean results are given in Fig. 4.

The reconstruction accuracy increases with the representation space size and saturates at a mean accuracy of about $\text {NCC}=0.70$, $\text {NCC}=0.72$ and $\text {NCC}=0.72$ for individual, generalized and generalized fine-tuned autoencoders, respectively, at a representation space size of 512. The reconstruction accuracy indicates that training was successful, but it does not give meaningful information about the tracking performance.

**Fig. 5:**
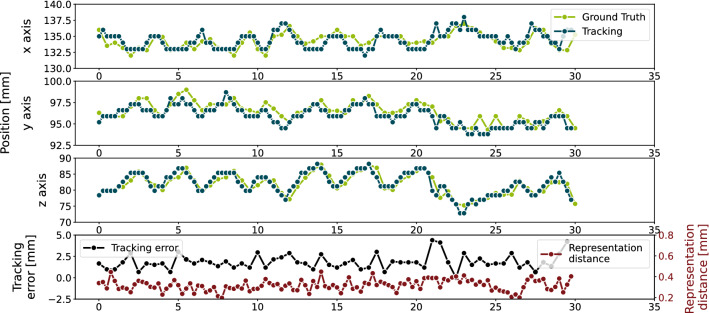
Tracking result (blue) using the autoencoder $AE_{genFT, 1}$ with representation space size of 256 and annotated ground truth (green) of the sequence $L_{1, 1}$ along the *x* (top), *y* (second) and *z* axis (third). On the bottom, the tracking error (black) measured by using L2-norm and the distance in the representation space (red) are presented. The mean tracking error of this example is 1.86 ± 0.81 mm

**Fig. 6:**
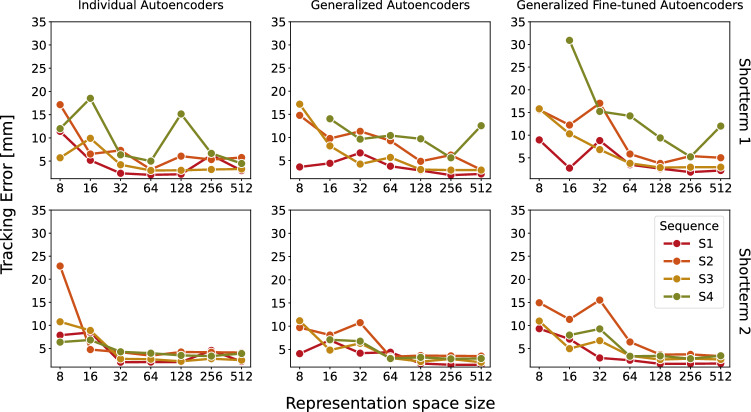
Tracking accuracy of the autoencoders $AE_{indiv, i}$ (left), $AE_{gen, i}$ (middle) and $AE_{genFT, i}$ (right) in the sequences $L_{1, i}$ (top) and $L_{2, i}$ (bottom) with different representation space sizes

Tracking is performed as described in “Landmark tracking” section. For evaluating the tracking accuracy, the tracking positions are compared to the ground truth landmarks using the L2-norm to determine the tracking error. In Fig. 5, the tracking result along all three axes in $L_{1, 1}$ with the autoencoder $AE_{genFT, 1}$ is illustrated. In this example the mean tracking error is 1.86 ± 0.81 mm. The mean tracking results of all experiments are illustrated in Fig. 6.

**Fig. 7:**
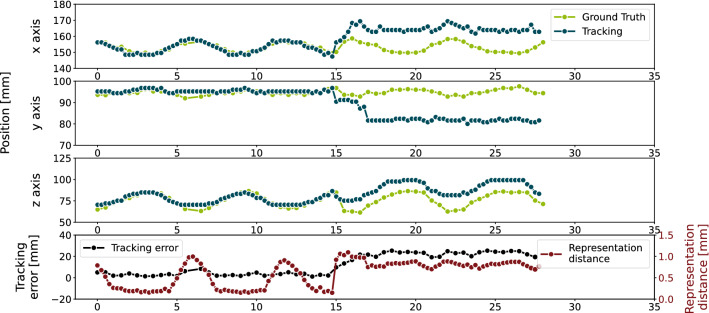
Tracking result (blue) using the autoencoder $AE_{genFT, 4}$ with representation space size of 512 and ground truth (green) of the sequence $L_{1, 4}$ along the *x* (top), *y* (second) and *z* axis (third). On the bottom, the tracking error (black) measured by using L2-norm and the distance in the representation space (red) are presented. The mean tracking error of this example is 12.54 ± 10.19 mm

At lower representation space sizes, the tracking error is high and it decreases when the representation space size is set higher. However, the tracking error saturates at a mean error of 4.47 ± 4.42 mm, 4.33 ± 5.14 mm and 3.23 ± 3.13 mm in individual, generalized and generalized fine-tuned autoencoders, respectively, at a representation space size of 512. While in the experiments with a representation space size smaller than 64 the tracking error is quite high, the experiments with the remaining representation space sizes the tracking algorithm was able to follow the target with a low position error. However, in Fig. 6 can be seen that some tracking experiments lead to a high tracking error. An example for these cases is given in Fig. 7 where the tracking result of the autoencoder $AE_{genFT, 4}$ with the representation space size of 512 applied in the sequence $L_{1, 4}$ is visualized. In this experiment, a tracking error of 12.54 ± 10.19 mm was measured. It can be seen that the algorithm loses the target after about 17 s and does never go back to it.

## Discussion

Deformable target tracking in 3D ultrasound is a challenging task due to low image quality, high noise ratio and the high degree of deformable target motion. In this study, an approach is presented to track a target patch in a representation space of a SWAE. Autoencoders are trained with different representation space sizes and in three different ways (individual, generalized, generalized fine-tuned). The reconstruction accuracy is evaluated, so it can be seen that all autoencoders converged during training. The results show that the reconstruction accuracy is in the same order of magnitude for all three kinds of autoencoders. This indicates that it is not necessary to train patient individual autoencoders, but a pretrained generalized autoencoder could be used. These results are promising concerning the clinical workflow since acquiring a mass of training data and training patient individual autoencoders is time consuming.

After training, the autoencoders were used to perform landmark tracking in representation space. The results indicate that the tracking algorithm is successful when using a representation space size larger than 64 and the error is not getting less when setting it very high as the error saturates after the representation space size of 256. The mean tracking errors of individual, generalized and generalized fine-tuned autoencoders indicate that the performances of individual and generalized autoencoders are comparable as the mean tracking error difference is 0.14 mm. However, using generalized autoencoders is beneficial because the mean tracking error decreases by 1.10 mm after fine-tuning the generalized autoencoders with small data sets. As seen in the reconstruction accuracy results, for the clinical workflow this is promising since for fine-tuning a pretrained generalized autoencoder acquiring a huge data set is not necessary. In addition, in the fine-tuning process only the fully connected layers are updated, so the computing time is reduced compared to training from scratch.

As can be seen in Fig. 7, in some experiments the tracking algorithm failed to follow the target at a certain point. This was always caused by position shifts of the annotated target which can be seen in the *z* axis in Fig. 7. The target shifts about 20 mm along the *z* axis between two annotated frames. Such shifts lead to tracking errors because the assumption that the next target position lies nearby the previous target position is violated. In these cases, the greedy search algorithm is not able to recover the target as it is a simple algorithm without any outlier handlings or regularizations. Apart form these cases, tracking with the greedy algorithm was successful which means that the target was not lost.

However, in some experiments the tracking error increased repeatedly in the same phase in the motion pattern. In Fig. 7, this can be seen at the time steps 0 s, 7 s and 12 s. The tracking algorithm is not able to find the exact target position in these phases. This is caused by the fact that only four fixed target references are used. In addition, these references are taken from a sequence that has a temporal distance to the tracked sequence. Although the motion is periodic, the target does not necessarily deform in the same way all the time. This means the target references taken from $L_{1, 1}$ can be inadequate for an exact tracking in $L_{2, 1}$. However, this approach is realistic as therapy planning is done some time before the treatment in the clinical workflow.

To the best of our knowledge, the presented method is the first unsupervised learning approach for target tracking in 4D ultrasound as well as the first approach that utilizes 3D Convolutional Neural Networks. In contrast, previously published approaches either use 2D slice images and are trained in a supervised fashion [11] or do not use Neural Networks [9, 10]. They achieve slightly smaller tracking errors on comparable but different data sets, ranging from 1.63 mm to 1.80 mm [11]. However, our unsupervised approach does not require a labeled data set, avoiding time-consuming manual annotations. Furthermore, we observed target loss errors caused by large target shifts as can be seen in Fig. 7. These errors could be avoided by improving the greedy search algorithm, so it might be possible to push the method’s accuracy even further.

It could be seen that tracking in representation space worked for all tested sequences. Since the presented method is based on autoencoders that were not trained for performing tracking but for simply reducing data dimensionality (Eq. 1), this is a promising result. This method works without the need for a huge labeled data set as an unsupervised learning approach is used. The tracking procedure can be performed using only four labeled target patches.

## Conclusion and future work

Target tracking in time resolved 3D ultrasound images is a challenging task. In this paper, a proof of concept for performing tracking in 3D ultrasound in a representation space generated by an SWAE is presented. It is a novel simple approach that uses a 3D convolutional network to perform target tracking in 3D ultrasound images. It could be shown that using a generalized autoencoder and fine-tuning it for a specific patient is more promising than training an individual autoencoder from scratch. In addition, it is shown that tracking in 3D ultrasound images can be performed without the need of a large labeled data set by using unsupervised representation learning. This approach holds the potential to learn the possible target deformations, e.g., by using deforming autoencoders [17] which could replace the need to define target references for the tracking. Thus, methods for learning the target shape variability will be investigated in future studies. Target tracking could be performed by using a simple greedy algorithm approach that is searching for a local optimum. However, in some experiments the target was lost due to large target shifts. Thus, the searching algorithm will be enhanced in future studies, e.g., by evaluating the representation distances. Furthermore, the needed representation space size is investigated and it could be shown that the tracking error saturates when increasing the representation space size. However, this is a first simple approach and it needs enhancements to make it more robust and to increase the tracking accuracy.
